# Supplementary material for: Zebrafish xenografts as a fast screening platform for bevacizumab cancer therapy
Source: Commun Biol. 2020 Jun 10;3:299. doi: 10.1038/s42003-020-1015-0 (PMC7286887; doi:10.1038/s42003-020-1015-0)
Supplement: Supplementary file 1 — Supplementary Information [file 42003_2020_1015_MOESM1_ESM.pdf]

Zebrafish xenografts as a fast screening platform for bevacizumab cancer therapy

SUPPLEMENTARY MATERIALS

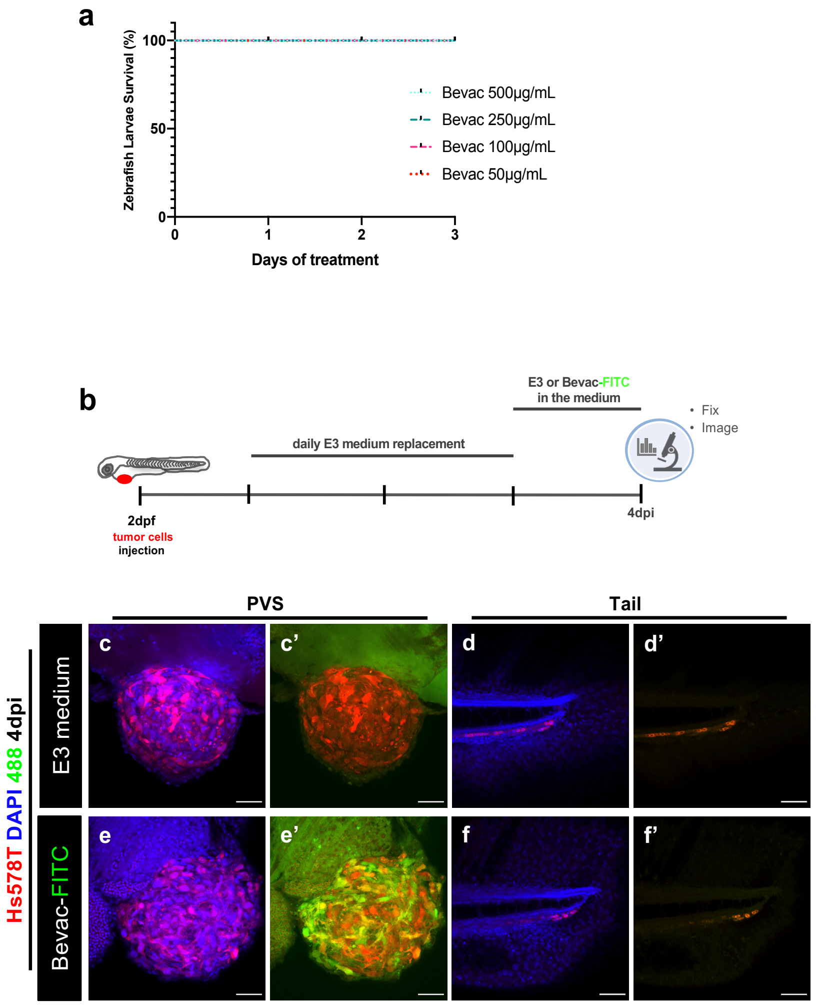

Supplementary Fig. 1| Concentration and diffusion of bevacizumab in zebrafish larvae. Zebrafish survival at different concentrations of bevacizumab in the fish water, N=50 larvae in each condition (**a**). Representative scheme of the protocol used to test the ability of zebrafish larvae xenograft model to absorb antibodies from the E3 medium (**b**). Hs578T cell line was fluorescently labelled with Cy5 (in red, false color) and injected into the PVS of 2dpf transparent zebrafish larvae and kept in E3 medium. At 3dpi, zebrafish xenografts were randomly distributed into two conditions: exposed to E3 medium (controls) or E3 medium supplemented with bevacizumab-FITC. At 4dpi, xenografts were stained with DAPI and imaged by confocal microscopy in two different sites: PVS (**c**, **c'**, **e**, **e'**) and tail (**d**, **d'**, **f, f'**). N=10, 2 independent experiments. Scale bars represent 50µm**.** All images are anterior to the left, posterior to right, dorsal up and ventral down.

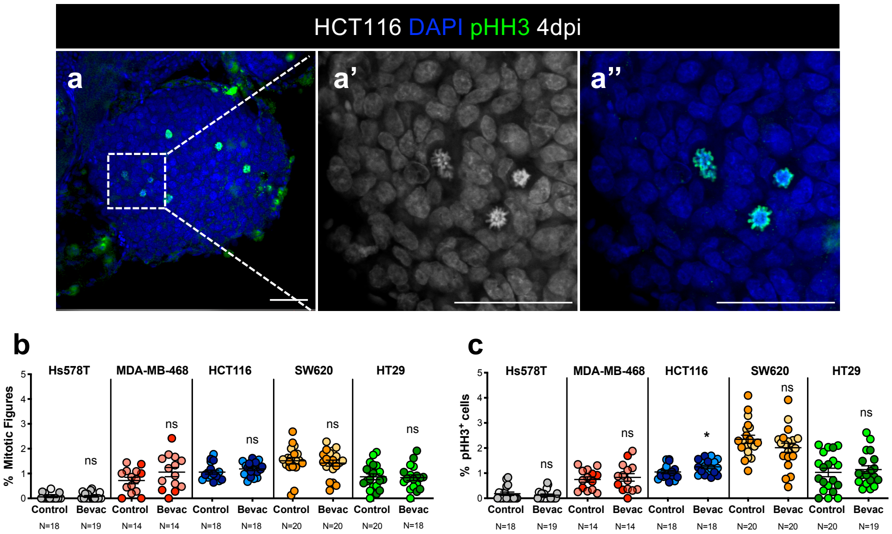

Supplementary Fig. 2| *In vivo* proliferation assessed in zebrafish xenografts by quantification of mitotic figures and pHH3. Human cancer cell lines (Hs578T, MDA-MB-468, HCT116, SW620 or HT29) were injected into the PVS of 2dpf *Tg(fli1:eGFP)* zebrafish larvae. Zebrafish xenografts were treated *in vivo* with bevacizumab and compared with untreated controls. At 4dpi, zebrafish xenografts were imaged by confocal microscopy (**a**-**a''**). The percentage of mitotic figures (**b**) and pHH3 (**c**, *P=0.0270) was quantified**.** The outcomes are expressed as AVG ± SEM. The number of xenografts analyzed are indicated in the graph and each dot represents one zebrafish xenograft. Results are from two independent experiments, which are highlighted in different colors corresponding to each individual experiment. Statistical analysis was performed using an unpaired *t-test*. Statistical results: (ns) > 0.05, *$P \leq 0.05$, **$P \leq 0.01$, ***$P \leq 0.001$, ****$P \leq 0.0001$. Scale bars represent 50μm. All images are anterior to the left, posterior to right, dorsal up and ventral down.

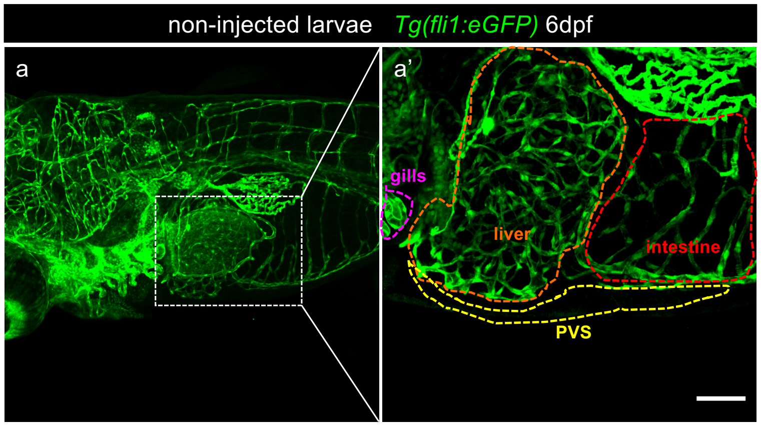

Supplementary Fig. 3| Vasculature of non-injected zebrafish larvae in the PVS region. Representative image of a non-injected *Tg(fli1:eGFP)* zebrafish larvae at 6 days post fertilization (equivalent of 4dpi) in the region of the PVS (a-a'). N=12 xenogratfs analyzed. Scale bars represent 50μm. All images are anterior to the left, posterior to right, dorsal up and ventral down.

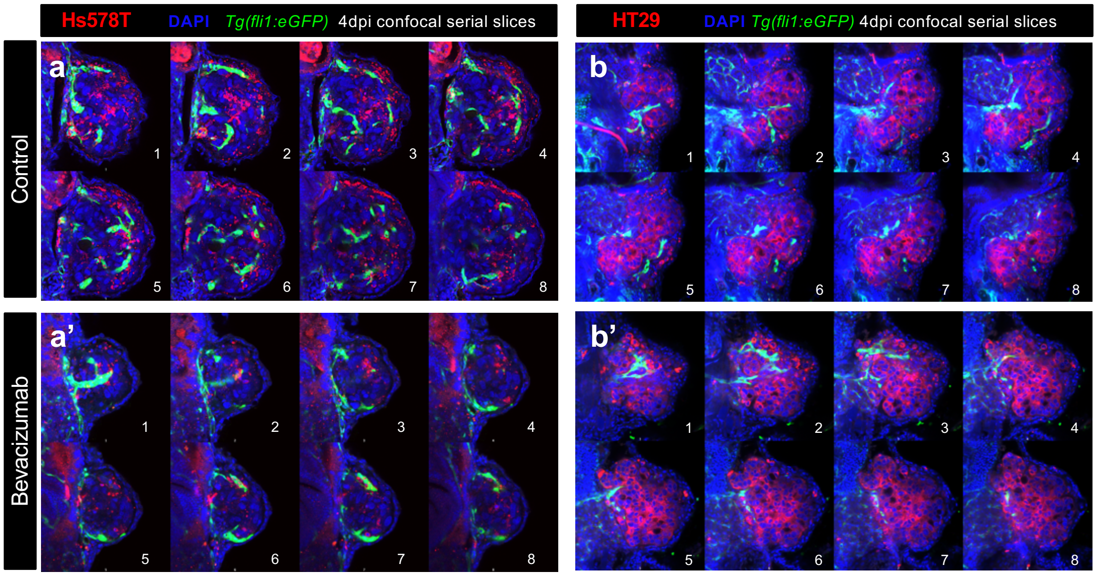

Supplementary Fig. 4| Examples of serial confocal slices of Hs578T and HT29 tumors. Human cancer cell lines (Hs578T and HT29) were fluorescently labelled with DiI (in red) and injected into the PVS of 2dpf *Tg(fli1:eGFP)* zebrafish larvae. Zebrafish xenografts were treated *in vivo* with bevacizumab and compared with untreated controls. At 4dpi, zebrafish xenografts were imaged by confocal microscopy every 5µm over a total of 40µm (**a-b'**). These are images representative of Figure 3.

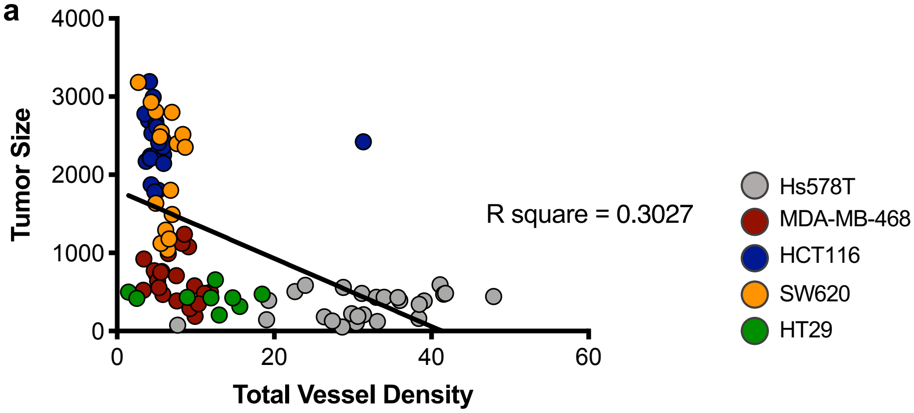

Supplementary Fig. 5| Correlation between tumor size and total vessel density in zebrafish xenografts. Scatter plot and linear regression between tumor size and the correspondent total vessel density in Hs578T, MDA-MB-468, HCT116, SW620 and HT29 tumors (**a**). Results are from two (Hs578T, MDA-MB-468, HCT116 and SW620) and one (HT29) independent experiments in a total of 98 xenografts analyzed.

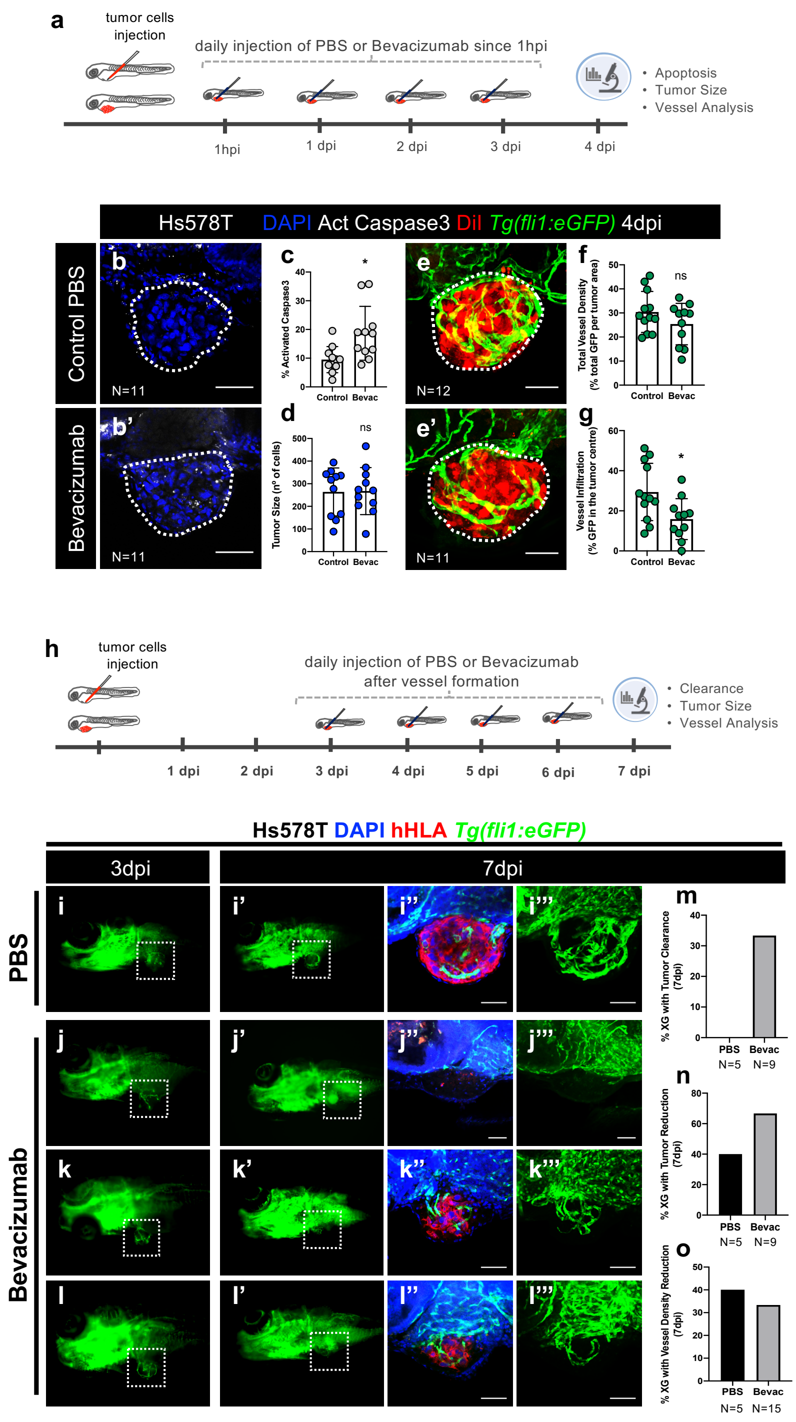

Supplementary Fig. 6| Daily injection of bevacizumab into circulation on Hs578T xenografts. a. Human cancer cell line Hs578T was injected into the PVS of 2dpf *Tg(fli1:eGFP)* zebrafish larvae. At 1 hour post injection (hpi), zebrafish xenografts were randomly distributed and either PBS or bevacizumab were injected into circulation. The procedure was repeated every 24h for the following three days. At 4dpi, zebrafish xenografts were sacrificed and analyzed by confocal microscopy (**a**, **b**', **b**, **e**, **e'**). The percentage of activated caspase3 (**c**, *P=0.0111), tumor size (**d**), total vessel density (**f**) and vessel infiltration (**g**, *P=0.0161) were calculated and the outcomes are expressed as AVG ± SEM. The number of xenografts analyzed are indicated in the representative images and each dot represents one zebrafish xenograft. **h.** Human cancer cell line Hs578T was injected into the PVS of 2dpf *Tg(fli1:eGFP)* zebrafish larvae. At 3dpi, zebrafish xenografts were randomly distributed and either PBS or bevacizumab were injected in circulation. The procedure was repeated every 24h for the following three days and xenografts were imaged daily. At 7dpi, xenografts were sacrificed and the tumor size and the vessel density of each xenograft was compared to the first day of treatment (**h**, **i-l'''**). The percentage of xenografts that presented tumor clearance (**m**), reduction of tumor volume (**n**) and reduction of vessel density (**o**) were calculated and the outcomes are expressed as AVG. The number of xenografts analyzed are indicated below the charts. Results are from 1 independent experiment in both settings, a and g. Statistical analysis was performed using an unpaired *t-test*. Statistical results: (ns) > 0.05, *P ≤ 0.05, **P ≤ 0.01, ***P ≤ 0.001, ****P ≤ 0.0001. Scale bars represent 50μm. All images are anterior to the left, posterior to right, dorsal up and ventral down.

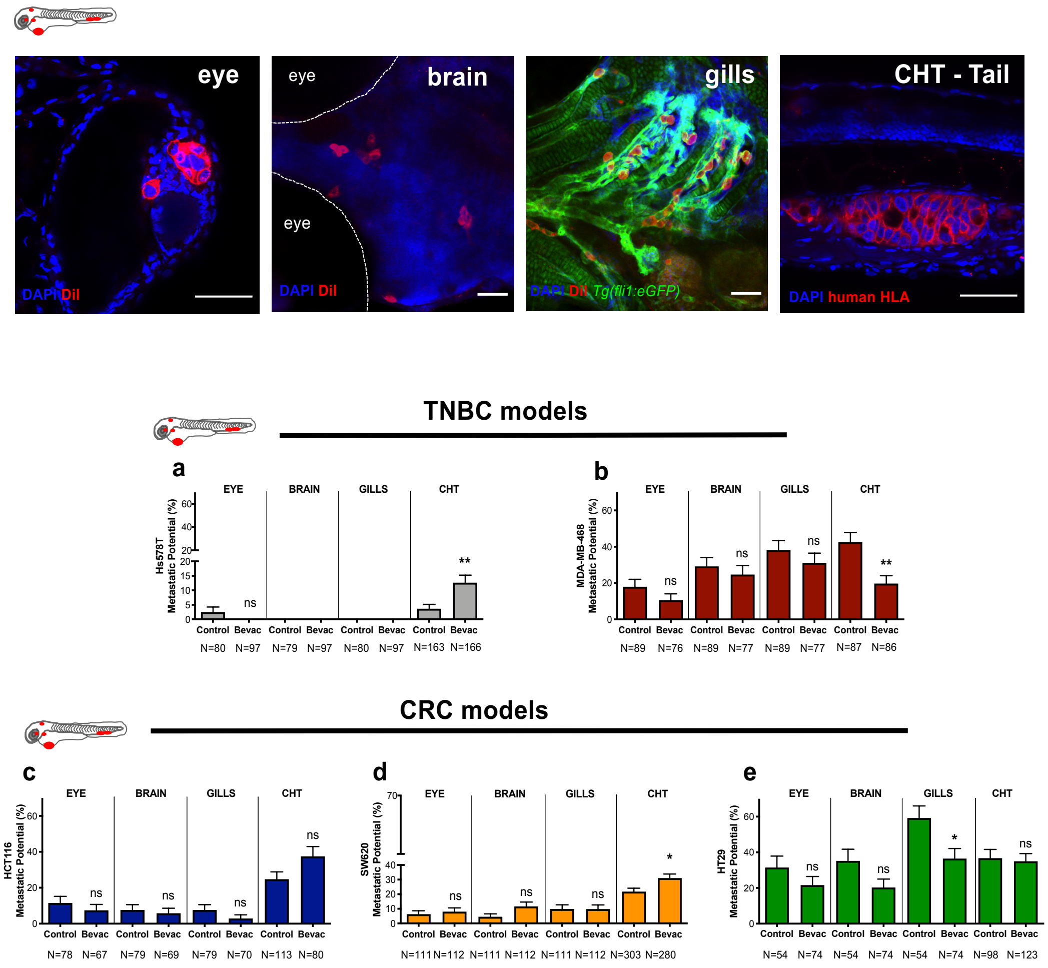

Supplementary Fig. 7| Distribution of micrometastasis in the different organs of zebrafish xenografts. Representative images of micrometastasis in the eyes, brain, gills and CHT. The percentage of xenografts that display micrometastasis in the different zebrafish body regions was quantified in each type of xenograft at 4dpi. Hs578T (**a**, **P=0.0042), MDA-MB-468 (**b**, **P=0.0017), HCT116 (**c**), SW620 (**d**, *P=0.0112) and HT29 xenografts (**e**, *P=0.0125). Results are from 4 (Hs578T), 3 (MDA-MB-468, SW620), 2 (HCT116) and 1 (HT29) independent experiments. The number of xenografts analyzed are indicated below the graphs. Outcomes are expressed as AVG ± SEM. Statistical analysis was performed using a Fisher's exact test. Statistical results: (ns) > 0.05, *P ≤ 0.05, ***P* ≤ 0.01, ****P* ≤ 0.001, *****P* ≤ 0.0001. Scale bars represent 50μm.

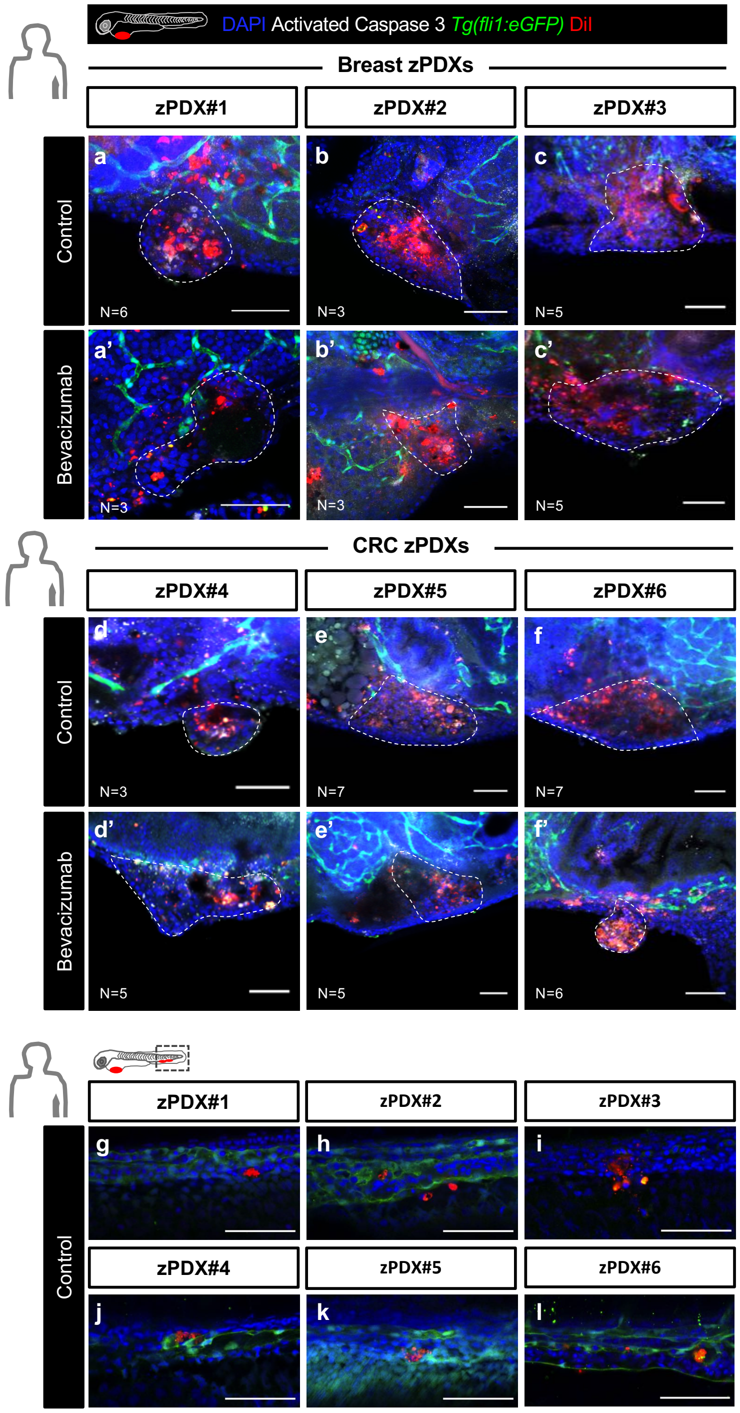

Supplementary Fig. 8| Human breast cancer and CRC surgical resected samples were injected into the PVS of 2dpf *Tg(fli1:eGFP)* zebrafish larvae. zPDXs were treated *in vivo* with bevacizumab and compared with untreated controls. At 4dpi, zebrafish xenografts were imaged by confocal microscopy to assess tumor size, apoptosis and tumor-induced angiogenesis (**a-f'**), The number of xenografts analyzed are depicted in the images. Representative confocal images of micrometastasis in the CHT from Figure 6 (**g-l**). Scale bars represent 50µm. All images are anterior to the left, posterior to right, dorsal up and ventral down.

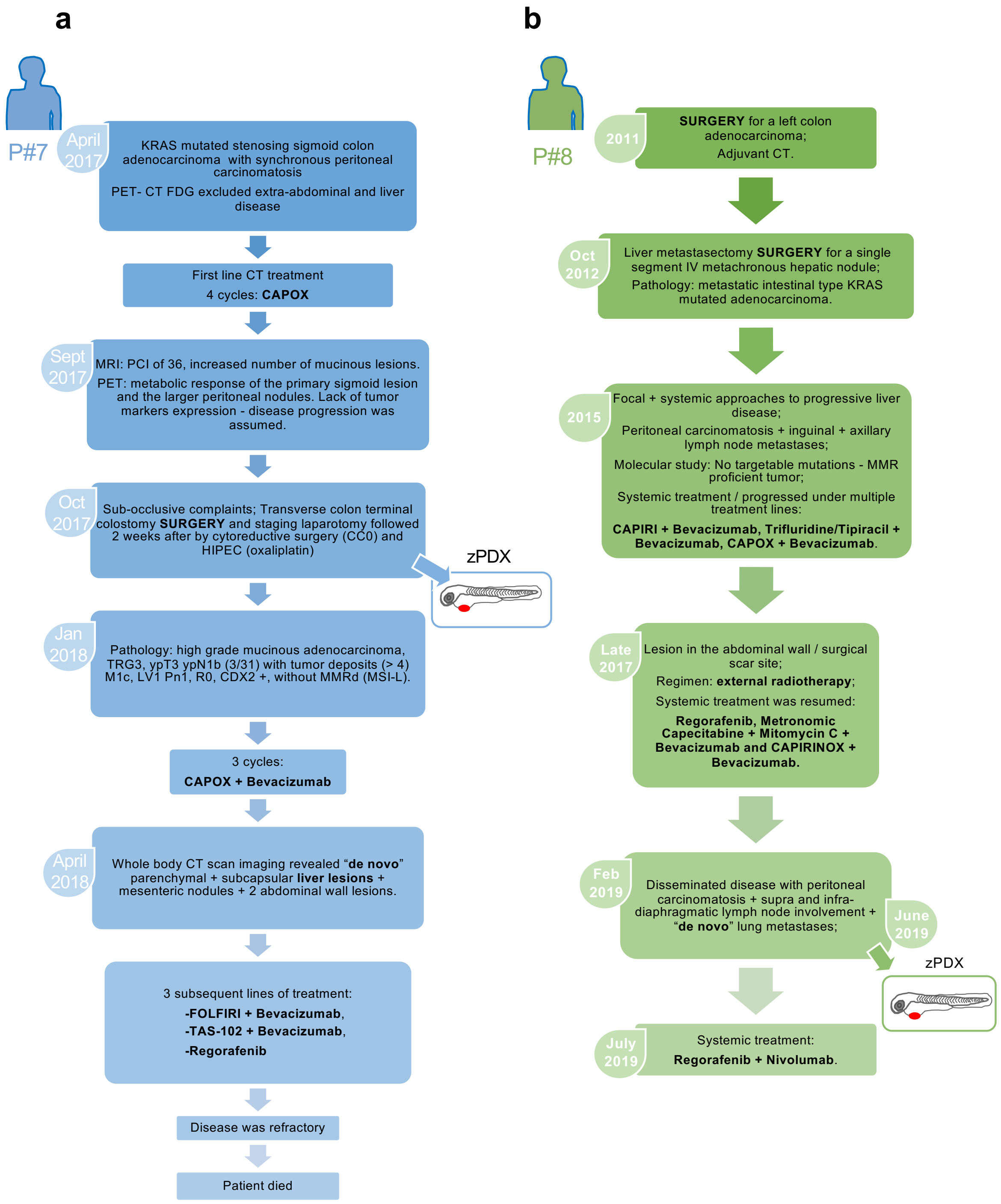
a
P#7
April 2017
KRAS mutated stenosing sigmoid colon adenocarcinoma with synchronous peritoneal carcinomatosis
PET- CT FDG excluded extra-abdominal and liver disease
First line CT treatment
4 cycles: CAPOX
Sept 2017
MRI: PCI of 36, increased number of mucinous lesions.
PET: metabolic response of the primary sigmoid lesion and the larger peritoneal nodules. Lack of tumor markers expression - disease progression was assumed.
Oct 2017
Sub-occlusive complaints; Transverse colon terminal colostomy SURGERY and staging laparotomy followed 2 weeks after by cytoreductive surgery (CC0) and HIPEC (oxaliplatin)
zPDX
Jan 2018
Pathology: high grade mucinous adenocarcinoma, TRG3, ypT3 ypN1b (3/31) with tumor deposits (> 4) M1c, LV1 Pn1, R0, CDX2 +, without MMRd (MSI-L).
3 cycles:
CAPOX + Bevacizumab
April 2018
Whole body CT scan imaging revealed "de novo" parenchymal + subcapsular liver lesions + mesenteric nodules + 2 abdominal wall lesions.
3 subsequent lines of treatment:
-FOLFIRI + Bevacizumab,
-TAS-102 + Bevacizumab,
-Regorafenib
Disease was refractory
Patient died
b
P#8
2011
SURGERY for a left colon adenocarcinoma;
Adjuvant CT.
Oct 2012
Liver metastasectomy SURGERY for a single segment IV metachronous hepatic nodule;
Pathology: metastatic intestinal type KRAS mutated adenocarcinoma.
2015
Focal + systemic approaches to progressive liver disease;
Peritoneal carcinomatosis + inguinal + axillary lymph node metastases;
Molecular study: No targetable mutations - MMR proficient tumor;
Systemic treatment / progressed under multiple treatment lines:
CAPIRI + Bevacizumab, Trifluridine/Tipiracil + Bevacizumab, CAPOX + Bevacizumab.
Late 2017
Lesion in the abdominal wall / surgical scar site;
Regimen: external radiotherapy;
Systemic treatment was resumed:
Regorafenib, Metronomic Capecitabine + Mitomycin C + Bevacizumab and CAPIRINOX + Bevacizumab.
Feb 2019
Disseminated disease with peritoneal carcinomatosis + supra and infra-diaphragmatic lymph node involvement + "de novo" lung metastases;
June 2019
zPDX
July 2019
Systemic treatment:
Regorafenib + Nivolumab.

Supplementary Fig. 9| Patient#7 and Patient#8 clinical history. Patient#7 is a 60-year-old male patient who was diagnosed with a KRAS mutated stenosing sigmoid colon adenocarcinoma in April/2017 with synchronous peritoneal carcinomatosis. Initial staging showed peritoneal lesions in all abdominal quadrants – magnetic resonance imaging PCI was 19. Positron emission tomography- computerized tomography FDG excluded extra-abdominal and liver disease. Started first line computerized tomography treatment with CAPOX regimen having completed 4 cycles. Despite the apparent decrease in the largest peritoneal lesions (solid cellular component), the MRI showed a PCI of 36 caused by the increase in the number of mucinous lesions. The PET demonstrated a metabolic response of the primary sigmoid lesion and the larger peritoneal nodules. At that time (September/2017) sub-occlusive complaints appeared and the patient was submitted to transverse colon terminal colostomy surgery and staging laparotomy followed 2 weeks after by cytoreductive surgery (CC0) and HIPEC (with Oxaliplatin). Pathology of the surgical specimen revealed a high grade mucinous adenocarcinoma, TRG3, ypT3 ypN1b (3/31) with tumor deposits (> 4) M1c, LV1 Pn1, R0, CDX2 +, without MMRd (MSI-L). In December/2017 postoperative computerized tomography was proposed with CAPOX + Bevacizumab which was started in late January/2018. Shortly after 3 cycles, whole body computerized tomography scan imaging revealed *"de novo"* parenchymal and subcapsular liver lesions, as well as mesenteric nodules and two abdominal wall lesions not previously present. Clinical and imaging progression was assumed. After three subsequent lines of treatment (FOLFIRI + Bevacizumab, TAS-102 + Bevacizumab, Regorafenib) to which the disease was refractory, the patient died (**a**).

Patient#8 is a 64-year-old man who underwent surgery for a left colon adenocarcinoma in 2011. In October 2012, 10 months after the end of adjuvant computerized tomography, he underwent liver metastasectomy surgery for a single segment IV metachronous hepatic nodule. Pathology report confirmed metastatic intestinal type KRAS mutated adenocarcinoma. In 2015, following various focal and systemic approaches to the progressive liver disease, the patient developed peritoneal carcinomatosis and inguinal and axillary lymph node metastases. An extensive molecular study did not show any targetable alteration and confirmed it as a MMR proficient tumor. The patient maintained systemic treatment and progressed under multiple treatment lines containing Bevacizumab (CAPIRI + Bevacizumab, Trifluridine/Tipiracil + Bevacizumab, CAPOX + Bevacizumab). In late 2017 he developed a lesion in the abdominal wall, at the surgical scar site, and underwent external radiotherapy. Systemic treatment was resumed and was sequenced with Regorafenib, Metronomic Capecitabine + Mitomycin C + Bevacizumab and CAPIRINOX + Bevacizumab, and progressive disease was documented with all of these regimens. In February/2019 image reassessment showed widely disseminated disease with peritoneal carcinomatosis, supra and infra-diaphragmatic lymph node involvement, and *"de novo"* lung metastases. The abdominal wall lesion was frankly ulcerated and biopsies were performed for the zebrafish study. Since July 2019, following further confirmation of disease progression, the patient resumed again systemic treatment with Regorafenib + Nivolumab **(b)**.

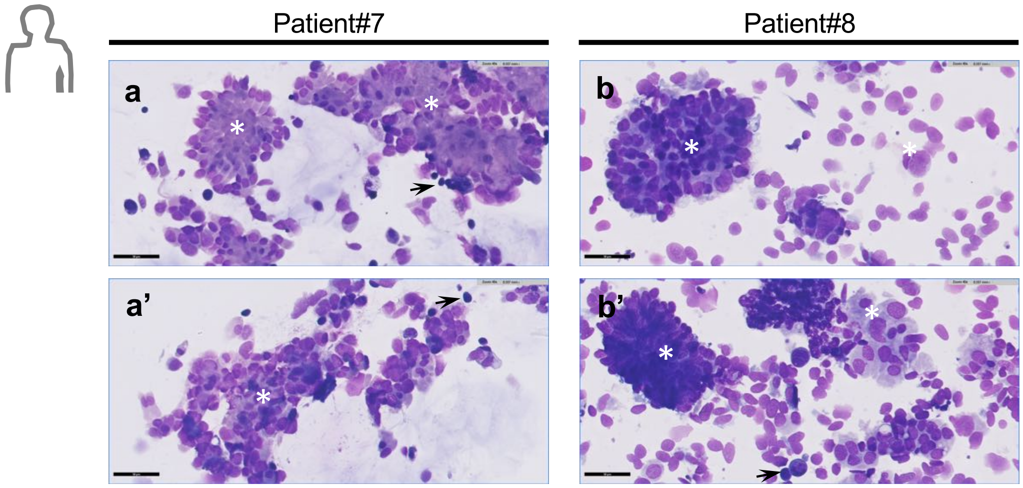

Supplementary Fig. 10| Giemsa-stained smears of tumor cell preparations prior to injection. Light micrographs of Giemsa-stained human samples from patient#7 (**a-a’**) and patient#8 (**b-b’**) prior to injection into the zebrafish larvae, showing a mixture of tumor cells and stroma. Giemsa-stained smears were produced to check adequacy/viability of the sample prior to injection. Upon microscopic analysis it is possible to observe a mixed population of tumor and supporting stroma and/or immune cell infiltrates, always with overrepresentation of neoplastic cells, as depicted by asterisks. For Patient#7, smears consist of numerous highly cohesive clusters of pleomorphic carcinomatous cells with scant cytoplasm (asterisk) and mixed with fewer immune cells (arrow). Patient#8 shows numerous highly to poorly cohesive clusters of pleomorphic carcinomatous cells (asterisk) and rare immune cells (arrow). Scale bars represent 50μm.

Supplementary Table 1. TNBC and CRC representative cell lines. Summary of the expression profile of each cancer cell line for the major and critical cancer-driven mutations, ligands and receptors of VEGF family, as well as sensitivity to bevacizumab described in the available literature.

	Hs578T	MDA-MB-468	HCT116	SW620	HT29	REF
**Origin**	Primary tumor	Pleural effusion metastasis	Primary tumor	Lymph node metastasis	Primary tumor	56
**Disease**	BC carcinoma	BC adenocarcinoma	CRC carcinoma	CRC adenocarcinoma	CRC adenocarcinoma	56
**KRAS**	*wt*	*wt*	G13D	G12V	*wt*	59–61
**PI3K**	*wt*	*wt*	H1047R	*wt*	P449T	38,61
**PTEN**	*wt*	Homo deletion	*wt*	*wt*	*wt*	38,61
**TP53**	V157F	R273H	*wt*	R273H P309S	R273H	61,62
**VEGF-A**	express	express	express	express	express	63–66
**VEGFR-2**	?	express	express	express	express	64–68
**NRP-1**	express	n. a	express	n. a	express	67,68
**VEGFR-1**	express	n. a	express	n. a	express	65,68
**Response to bevacizumab**	n. a	resistant	n. a	n. a	resistant	45,46
**Animal model tested**		mouse			mouse	45,46
**Metastatic potential**	clones diff. potential	highly metastatic	metastatic	metastatic	highly metastatic	53–55

Supplementary Table 2 – List of patient samples used to generate zPDX. List of surgically resected human breast and rectum and metastasis of CRC samples. Tumor node metastasis status: T1, tumor invades submucosa; pN0, no malignant lymph nodes; pN1, cancer cells are found in one to three lymph nodes; pN1a, cancer cells in one regional lymph node; pN1b, cancer cells in two to three regional lymph nodes; pN2, cancer cells in four or more regional lymph nodes; pN2a, cancer cells are found in four to six lymph nodes.

Patient sample	Primary tumor location	stage
**P1**	Breast carcinoma – Luminal Her2-	cT1cN0M0
**P2**	Breast carcinoma – Ductal Her2+	pT1cN0(sn)M0
**P3**	Breast carcinoma – Luminal Her2-	cT1cN0M0
**P4**	Liver metastasis from CRC	N/A
**P5**	Rectum	T1N1
**P6**	Liver metastasis from CRC	N/A
**P7**	Adenocarcinoma	TRG3, ypT3 ypN1b
**P8**	Skin metastasis biopsy from metastatic intestinal adenocarcinoma	N/A

Supplementary Table 3. Reagents/solutions for patient tissue sample processing.

		Reagent	Supplier	Cat. Number	Final Concentration
**CRC samples**	Mix1	DMEM/F-12	Gibco	11320-074	—
		FBS	Gibco	10270106	40%
		Y-27632	Cliniscience	A11001	10 μM
		Primocin	Invivogen	ANT-PM-2	100 μg/mL
		Putrescin	Sigma-Aldrich	P5780	10 μg/mL
		Nicotinamide	Sigma-Aldrich	N3376	10 mM
	Mix2	DMEM/F-12	Gibco	11320-074	—
		Y-27632	Cliniscience	A11001	10 μM
		Primocin	Invivogen	ANT-PM-2	100 μg/mL
		Putrescin	Sigma-Aldrich	P5780	10 μg/mL
		Nicotinamide	Sigma-Aldrich	N3376	10 mM
		DNAse I	Fermentas	EN0525	5 U/mL
**Breast samples**	Mix1	DMEM/F-12	Gibco	11320-074	—
		FBS	Gibco	10270106	60%
		Y-27632	Cliniscience	A11001	10 μM
		Primocin	Invivogen	ANT-PM-2	100 μg/mL
		Putrescin	Sigma-Aldrich	P5780	10 μg/mL
		Nicotinamide	Sigma-Aldrich	N3376	10 mM
		Gentamicin	Sigma-Aldrich	G1397	50 μg/mL
		Hydrocortisone	Sigma-Aldrich	H0888	0.5 μg/mL
		Insulin	Sigma-Aldrich	I6634	5 μg/mL
		Glutamax	Gibco	35050038	1X
	Mix2	DMEM/F-12	Gibco	11320-074	—
		Y-27632	Cliniscience	A11001	10 μM
		Primocin	Invivogen	ANT-PM-2	100 μg/mL
		Putrescin	Sigma-Aldrich	P5780	10 μg/mL
		Nicotinamide	Sigma-Aldrich	N3376	10 mM
		Gentamicin	Sigma-Aldrich	G1397	50 μg/mL
		Hydrocortisone	Sigma-Aldrich	H0888	0.5 μg/mL
		Insulin	Sigma-Aldrich	i6634	5 μg/mL
		Glutamax	Gibco	35050038	1X
