# Supplementary material for: Zebrafish xenografts as a fast screening platform for bevacizumab cancer therapy
Source: Commun Biol. 2020 Jun 10;3:299. doi: 10.1038/s42003-020-1015-0 (PMC7286887; doi:10.1038/s42003-020-1015-0)
Supplement: Supplementary file 9 — Description of Additional Supplementary Files [file 42003_2020_1015_MOESM9_ESM.pdf]

Description of Additional Supplementary Files

File Name: **Supplementary Movie 1**
Description: Confocal Z stack of the vasculature of Hs578T control xenografts at 4dpi. DAPI in blue, tumor cells in red (DiI) and vasculature in green *Tg(fli1:eGFP)*.

File Name: **Supplementary Movie 2**
Description: Confocal Z stack of Hs578T xenografts at 4dpi, treated with bevacizumab, see reduction of infiltrated vessels. DAPI in blue, tumor cells in red (DiI) and vasculature in green *Tg(fli1:eGFP)*.

File Name: **Supplementary Movie 3**
Description: Confocal Z stack of HT29 control xenografts at 4dpi in *Tg(fli1:eGFP)* hosts. DAPI in blue, tumor cells in red (DiI) and vasculature in green *Tg(fli1:eGFP)*.

File Name: **Supplementary Movie 4**
Description: Confocal Z stack of HT29 tumors at 4dpi, treated with bevacizumab. DAPI in blue, tumor cells in red (DiI) and vasculature in green *Tg(fli1:eGFP)*.

File Name: **Supplementary Data 1**

Description: All source data underlying the graphs presented in the main figures, each sheet has the raw data corresponds to each main figure
