# Supplementary material for: Zebrafish xenografts as a fast screening platform for bevacizumab cancer therapy
Source: Commun Biol. 2020 Jun 10;3:299. doi: 10.1038/s42003-020-1015-0 (PMC7286887; doi:10.1038/s42003-020-1015-0)
Supplement: Supplementary file 10 — Reporting Summary [file 42003_2020_1015_MOESM10_ESM.pdf]

nature research

Corresponding author(s): RITA FIOR

Last updated by author(s): 24/03/2020

Reporting Summary

Nature Research wishes to improve the reproducibility of the work that we publish. This form provides structure for consistency and transparency in reporting. For further information on Nature Research policies, see Authors & Referees and the Editorial Policy Checklist.

Statistics

For all statistical analyses, confirm that the following items are present in the figure legend, table legend, main text, or Methods section.

n/a	Confirmed	
☐	☒	The exact sample size (n) for each experimental group/condition, given as a discrete number and unit of measurement
☐	☒	A statement on whether measurements were taken from distinct samples or whether the same sample was measured repeatedly
☐	☒	The statistical test(s) used AND whether they are one- or two-sided<br>*Only common tests should be described solely by name; describe more complex techniques in the Methods section.*
☒	☐	A description of all covariates tested
☐	☒	A description of any assumptions or corrections, such as tests of normality and adjustment for multiple comparisons
☐	☒	A full description of the statistical parameters including central tendency (e.g. means) or other basic estimates (e.g. regression coefficient) AND variation (e.g. standard deviation) or associated estimates of uncertainty (e.g. confidence intervals)
☐	☒	For null hypothesis testing, the test statistic (e.g. F, t, r) with confidence intervals, effect sizes, degrees of freedom and P value noted<br>*Give P values as exact values whenever suitable.*
☒	☐	For Bayesian analysis, information on the choice of priors and Markov chain Monte Carlo settings
☒	☐	For hierarchical and complex designs, identification of the appropriate level for tests and full reporting of outcomes
☒	☐	Estimates of effect sizes (e.g. Cohen's d, Pearson's r), indicating how they were calculated

Our web collection on statistics for biologists contains articles on many of the points above.

Software and code

Policy information about availability of computer code

Data collection	Zeiss Zen software from Zeiss710 confocal microscope and Image J software
Data analysis	Prism8 software and Excell

For manuscripts utilizing custom algorithms or software that are central to the research but not yet described in published literature, software must be made available to editors/reviewers. We strongly encourage code deposition in a community repository (e.g. GitHub). See the Nature Research guidelines for submitting code & software for further information.

Data

Policy information about availability of data

All manuscripts must include a data availability statement. This statement should provide the following information, where applicable:

- Accession codes, unique identifiers, or web links for publicly available datasets
- A list of figures that have associated raw data
- A description of any restrictions on data availability

The data that support the findings of this study are available from the corresponding author upon reasonable request.

Field-specific reporting

Please select the one below that is the best fit for your research. If you are not sure, read the appropriate sections before making your selection.

☒ Life sciences ☐ Behavioural & social sciences ☐ Ecological, evolutionary & environmental sciences

Life sciences study design

All studies must disclose on these points even when the disclosure is negative.

Sample size	No sample-size calculation was performed.<br>To quantify proliferation, apoptosis induction and tumor size and vessel density we analized confocal images acquired in the Zeiss -710. The minimum number of larvae analyzed per independent experiment was 22 and maximum was 48.<br>For metastasis, we quantified the incidence of fish with micrometastais per experimental condition, the N varies between 28 and 303.
Data exclusions	We only excluded 2 values - statistical outliers, determined by GraphPad QuickCalcs: outlier calculator in Fig 3f-g
Replication	To verify the reproducibility of the experimental findings, all the experiments were repeated at least two times.<br>The number of independent experiments realized for every cell line and figure is present in the corresponding legend.
Randomization	Before injection, we mix a pool of zebrafish larvae and anesthetize them.<br>In the moment of injection, we randomly take some larvae and inject two conditions - control (cells non-resuspended in Bevacizumab) and experimental condition (cells resuspended in Bevacizumab).
Blinding	In general the work was done from the beginning to the end by only one researcher, therefore making it very difficult to do blind analysis. Although the work started by Raquel Mendes (RM), then RM had to focus on another project and so Catia Rebelo de Almeida did most of the work, after validating RM first results.

Reporting for specific materials, systems and methods

We require information from authors about some types of materials, experimental systems and methods used in many studies. Here, indicate whether each material, system or method listed is relevant to your study. If you are not sure if a list item applies to your research, read the appropriate section before selecting a response.

Materials & experimental systems

n/a	Involved in the study	
☐	☒	Antibodies
☐	☒	Eukaryotic cell lines
☒	☐	Palaeontology
☐	☒	Animals and other organisms
☐	☒	Human research participants
☐	☒	Clinical data

Methods

n/a	Involved in the study	
☒	☐	ChIP-seq
☒	☐	Flow cytometry
☒	☐	MRI-based neuroimaging

Antibodies

Antibodies used	Anti-Cleaved Caspase 3 - supplier Cell Signaling - catalog#9661 - clone name Asp175 - lot number<br>Anti-GFP - supplier Roche - catalog#11814460001 - clone number 7.1/13.1 monoclonal, anti-phospho Histone H3 rabbit Milipore catalog#06-570; Anti-human HLA (rabbit former MHC-class I ABCAM, ab52922).
Validation	Anti-Cleaved Caspase 3 was validated by KO validation (see Cell Signalling website). We used anti-GFP mostly to enhance expression of the GFP from the transgenic and were able to detect GFP with no problem.

Eukaryotic cell lines

Policy information about cell lines

Cell line source(s)	Hs578T , MDA-MB-468, SW620 and HT-29 were derived from American Type Culture Collection and provided by Mónica Bettencourt Dias from Instituto Gulbenkian da Ciência. HCT116 cell line was derived from American Type Culture Collection and provided by Dr. Ângela Relógio from Charité Medical University of Berlim.
Authentication	All cell lines were authenticated through short tandem repeat (STR) profile analysis.
Mycoplasma contamination	All cell lines were tested and negative for mycoplasma contamination.
Commonly misidentified lines<br>(See ICLAC register)	The study did not involve misidentified lines.

Animals and other organisms

Policy information about studies involving animals; ARRIVE guidelines recommended for reporting animal research

Laboratory animals	In vivo experiments were performed in zebrafish model (Danio rerio), which was maintained and handled in accordance with European Animal Welfare Legislation, Directive 2010/63/EU and Champalimaud Fish Platform Program.<br>Adult zebrafish were kept in 3.5L tanks with a maximum population of 30 fish per tank, both male and female, in a running water system, feeded twice a day and maintained in a temperature and humidity controlled environment, as well as, day-night automatic cycle of 14 hours light plus 10 hours dark.<br>Adults were used to breed and the experiments were performed in 2dpf zebrafish larvae.
Wild animals	The study did not involve wild animals.
Field-collected samples	The study did not involve samples collected from the field.
Ethics oversight	Portuguese institutional organizations- ORBEA (Órgão de Bem-Estar e Ética Animal / Animal Welfare and Ethics Body) and DGAV (Direção Geral de Alimentação e Veterinária / Directorate General for Food and Veterinary) approved this study and corresponding protocols.

Note that full information on the approval of the study protocol must also be provided in the manuscript.

Human research participants

Policy information about studies involving human research participants

Population characteristics	N/a we had access to very small number of patients so we could not discriminate in different groups.
Recruitment	The project was explained to patients from Hospital Professor Doutor Fernando Fonseca and Centro Clínico Champalimaud and patients become as participants after signing an informed consent if interested in joining the study.
Ethics oversight	The ethics committee of Hospital Professor Doutor Fernando Fonseca and Champalimaud Foundation approved this project.

Note that full information on the approval of the study protocol must also be provided in the manuscript.

Clinical data

Policy information about clinical studies

All manuscripts should comply with the ICMJE guidelines for publication of clinical research and a completed CONSORT checklist must be included with all submissions.

Clinical trial registration	NA
Study protocol	The ethics committee of Hospital Professor Doutor Fernando Fonseca and Champalimaud Foundation approved this project.
Data collection	The project was explained to patients from Hospital Professor Doutor Fernando Fonseca and Centro Clínico Champalimaud and patients become as participants after signing an informed consent if interested in joining the study. We had access to patient data : age, sex, diagnosis, biopsy/surgery pathology reports, CT scans and clinical history.
Outcomes	Clinical outcome was defined by CT scans and pathology progression
